# GeneSippr: A Rapid Whole-Genome Approach for the Identification and Characterization of Foodborne Pathogens such as Priority Shiga Toxigenic *Escherichia coli*

**DOI:** 10.1371/journal.pone.0122928

**Published:** 2015-04-10

**Authors:** Dominic Lambert, Catherine D. Carrillo, Adam G. Koziol, Paul Manninger, Burton W. Blais

**Affiliations:** Research and Development, Ottawa Laboratory Carling, Science Branch, Canadian Food Inspection Agency, Ottawa, Ontario, Canada; USDA-ARS-ERRC, UNITED STATES

## Abstract

The timely identification and characterization of foodborne bacteria for risk assessment purposes is a key operation in outbreak investigations. Current methods require several days and/or provide low-resolution characterization. Here we describe a whole-genome-sequencing (WGS) approach (GeneSippr) enabling same-day identification of colony isolates recovered from investigative food samples. The identification of colonies of priority Shiga-toxigenic *Escherichia coli* (STEC) (i.e., serogroups O26, O45, O103, O111, O121, O145 and O157) served as a proof of concept. Genomic DNA was isolated from single colonies and sequencing was conducted on the Illumina MiSeq instrument with raw data sampling from the instrument following 4.5 hrs of sequencing. Modeling experiments indicated that datasets comprised of 21-nt reads representing approximately 4-fold coverage of the genome were sufficient to avoid significant gaps in sequence data. A novel bioinformatic pipeline was used to identify the presence of specific marker genes based on mapping of the short reads to reference sequence libraries, along with the detection of dispersed conserved genomic markers as a quality control metric to assure the validity of the analysis. STEC virulence markers were correctly identified in all isolates tested, and single colonies were identified within 9 hrs. This method has the potential to produce high-resolution characterization of STEC isolates, and whole-genome sequence data generated following the GeneSippr analysis could be used for isolate identification in place of lengthy biochemical characterization and typing methodologies. Significant advantages of this procedure include ease of adaptation to the detection of any gene marker of interest, as well as to the identification of other foodborne pathogens for which genomic markers have been defined.

## Introduction

Traditional techniques for the detection of pathogenic bacteria in foods rely on a multi-step process involving pre-enrichment in a selective broth, followed by plating to obtain colony isolates, which are then purified and subjected to a battery of biochemical and serological tests to confirm their identity. The process of definitively identifying bacterial colonies on primary isolation plates can take up to one week to complete because of the requirement for growth and expression of phenotypic characteristics specific to the organism. In some cases (e.g., detection of Shiga-toxigenic *E*. *coli* (STEC) of public health concern), phenotypic methods are entirely impractical as a means of identification. Ultimately, these techniques are limited in terms of the type of information (e.g., risk profiling) that can be garnered from an isolate to underscore risk management decisions.

STEC infections can result in serious medical conditions including bloody diarrhea, hemolytic-uremic syndrome (HUS), kidney failure, microangiopathic hemolytic anemia, and can occasionally be fatal. There are no biochemical features by which most so-called priority STEC strains can be differentiated from commensal *E*. *coli* or other STEC which are not a public health concern. However, it is universally recognized that foodborne STEC posing a public health risk can be defined on the basis of certain gene markers, including the Shiga-toxin genes, *stx1* or *stx2*, the intimin-coding gene, *eae*, and markers for the specific serogroups of concern (e.g., O26, O45, O103, O111, O121, O145 and O157) [1–3]. The STEC method utilized by the Canadian Food Inspection Agency [1, 2] features a PCR procedure (EHEC-7 CHAS) for the identification of colony isolates on the basis of these defining gene markers within one work day [4]. “Positive” primary isolates are shipped thereafter to a specialized typing laboratory for further analysis by multiple-locus variable number tandem repeat analysis (MLVA) and pulsed-field gel electrophoresis (PFGE), a process that requires several days and incurs delays in the resolution of outbreak investigations. PCR techniques also have their limitations: primers and amplification conditions require extensive optimization, and whenever the definition of a pathogen group (e.g., priority STEC) changes to reflect public health trends (e.g., emergence of new priority O serogroups and virulence factors, such as *AggR* or *AaiC*) it is necessary to re-develop and validate the PCR primers and conditions.

**Fig 1:**
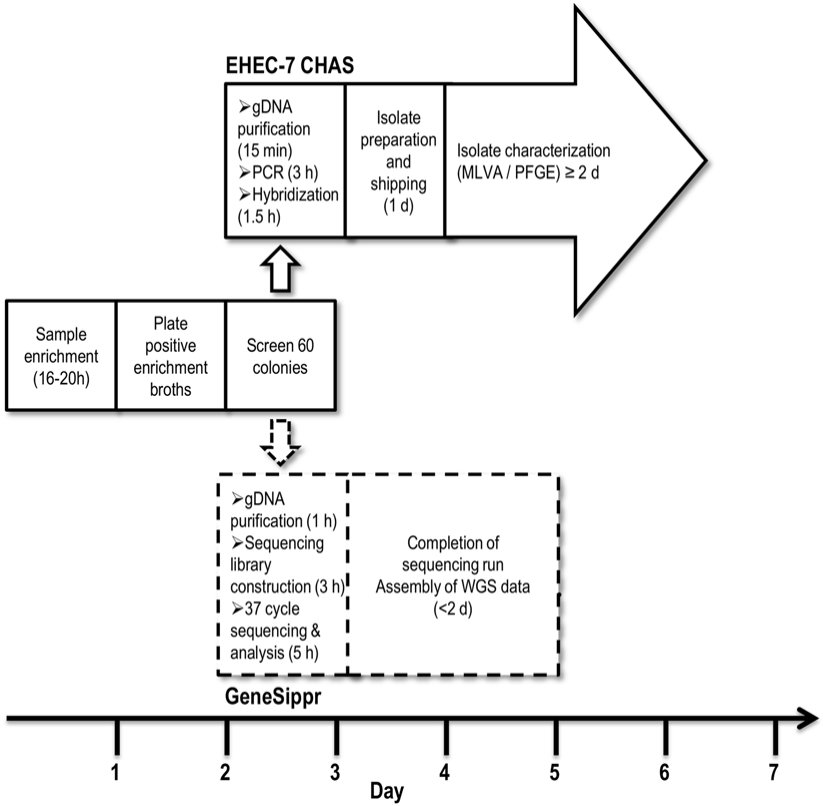
Timelines for detection of STEC in food testing laboratories. In the standard approach (Days 1 to 3), samples taken from foods (e.g., ground beef) are added to enrichment broths developed to favor growth of STEC. Following the enrichment procedure, broth cultures are screened for STEC by PCR, and positive samples are plated on agar media [4]. On the third day, putative STEC colonies are identified by PCR screening. The EHEC-7 CHAS (top line) is used to confirm presence of genomic targets identifying colonies as STEC (e.g., O-type, Shiga-toxin, *eae*) and confirmed priority STEC are shipped to specialized facilities for typing by MLVA and/or PFGE. In the GeneSippr approach (lower line), presumptively positive STEC colonies are identified by whole genome sequencing within similar time frames as the standard method. Following the completion of the sequencing run, whole genome sequence (WGS) data could be assembled and/or shared with public health agencies for use in high-resolution typing methods such as whole-genome MLST (wgMLST).

Leading-edge genomic technologies open new possibilities for comprehensive analyses of microbial isolates recovered from food samples. Next-generation sequencing (NGS) technologies can now render a bacterial genome much faster and at a significantly lower cost than previously possible. The value of rapid benchtop sequencing in the investigation of foodborne disease outbreaks is becoming increasingly accepted [5–12]. Implementing NGS capacity in analytical laboratories supporting food inspection programs would generate high-resolution strain characterization enabling unambiguous identification of pathogens, facilitate detection of relevant genetic markers underpinning the development of risk profiles, eliminate delays associated with shipping isolates to typing facilities, and provide a one-method-fits-all solution for the identification of food pathogens. One limitation of NGS sequencing is the time required (>2 days) for completion of a full sequencing run. To be suitable for use in a food testing laboratory, the total time frame for sample preparation (preferably from a primary colony isolate) and data acquisition should be within the range of current analytical approaches such as the EHEC-7 CHAS procedure used to identify foodborne colony isolates [3,4] (i.e., within one working day) (Fig 1).

We set out to determine if NGS could be adapted to same-day identification and characterization of bacterial isolates recovered from foods while allowing the sequencing reaction to continue uninterrupted until completion. NGS data so generated could be analyzed almost immediately to support timely risk management actions without impacting the quality of whole-genome sequencing (WGS) data generated following the analysis. Here we present GeneSippr, a novel genomic tool for the analysis of single colony isolates based on the sampling (or “sipping”) of raw data during the early stages of the WGSprocess to determine the presence of pre-defined gene markers, and evaluate a set of quality metrics designed to validate the analysis.

This new approach may be regarded as an identification system providing ultimate multiplexing capacity in terms of the number of genomic markers which can be interrogated in a single procedure. Such an algorithmic approach has the inherent flexibility to enable determination of the presence of any genomic marker of interest on an *ad hoc* basis. For example, in the course of a food safety investigation information may come to light which would prompt further investigation of genomic markers (e.g., virulence or antimicrobial resistance genes) to inform a risk management decision. Conventional tools such as PCR do not allow such *ad hoc* determinations within the time course of a food safety investigation because of the need to optimize and validate each new primer added to a reaction system. The priority STEC constitute a striking example of how genomic technologies can be used to discern the presence of gene markers pinpointing a family of pathogens otherwise not readily amenable to identification by classic means. For demonstration purposes, we focussed on the adaptation of GeneSippr to the identification of priority STEC in an approach modeling the previously described EHEC-7 CHAS method [4] (Fig 1).

## Materials and Methods

### Target identification and preparation

Two categories of gene targets were used in this study: 1) EHEC-7 targets, and 2) genomically dispersed conserved sequence (GDCS) quality control targets. The unique, diagnostic sequences used to detect these gene targets *in silico* are referred to as e-probes. The EHEC-7 e-probes used in this study correspond to the amplicon-specific oligonucleotide capture probes currently used in the detection of priority STEC at the Canadian Food Inspection Agency (Table 1) [4, 13, 14]. The GDCS e-probes were designed by identifying regions of approximately 50 nt with qualitatively high levels of sequence identity in multiple sequence alignments (ClustalX) [15] of alleles derived from 15 housekeeping genes comprising the Pasteur [16] and Achtman [17] *E*. *coli* multilocus sequence typing (MLST) schemes. An e-probe created in the same manner from a housekeeping gene from *Campylobacter jejuni*, *aspA* [18], and the sequence of a synthetic construct, MyIC, [19] were included as negative control features.

**Table 1:** Sequences of e-probes used in this study.

Reference	Gene target	e-probe Sequence
EHEC-7 CHAS [4, 14]	*wzx*, O26	AATTAGAACCATACAAAGTTGGAGAATATAAAAGCCTGCTATATGCAAGC
	*wzx*, O45	TACGTCTGGCTGCAGGGACTTTCGTTGCGTTGTGCATGGTGGCATGGG
	*wzx*, O103	CGAATGTTTTAGCCATATCCTCATCGTTGTTATCTATGGTGGGCTTAGTT
	*wzx*, O111	TCTTGTATGTCTGAATATTACCGGTTGTTTCATCAATCCTAATTTTAATA
	*wzx*, O121	GGTCGTGAAACAGCTCGCTATCATGGCGGGACAATGACAGTGCTGGACTACA
	*wzx*, O145	TTTGGTTTGGTGGTACTGTGTCCGCGAGTGTGCTTGGAGTGGCTTATATT
	*rfbE*, O157	TAAAACTATTACTACAGGTGAAGGTGGAATGGTTGTCACGAATGACAAAA
	*stx*_*1*_	ACTGGATGATCTCAGTGGGCGTTCTTATGTAATGACTGCTGAAGATGTTG
	*stx*_*2*_	CACATATATCAGTGCCCGGTGTGACAACGGTTTCCATGACAACGGACAGC
	*eae*	ACAGTTCCGAAAGCGAAATGATGAAGGCTGGACCTGGTCAGCAGATCATT
GDCS^a^ MLST (Achtman) [17]	*adk*	AAGGGACTCAGGCTCAGTTCATCATGGAGAAATATGGTATTCCGCA
	*fumC*	ATGGAACGTAAAGTTCACTCTAACGACGACGTGAACAAAAGCCAAAG
	*gyrB*	GGTCTGCACGGCGTTGGTGTTTCGGTAGTAAACGCCCTGTCGCAAAAACTGGA
	*icd*	GAGCGTAAAATCTCCTGGATGGAAATTTACACCGGTGAAAAATCCACACAGGTTTATGGTCA
	*mdh*	GCGCGTAAACCGGGTATGGATCGTTCCGACCTGTTTAACGTTAACG
	*purA*	GCACGTCGCGGTCTGCGTGTTGGCGACCTTTTCGACAAAGAAACCTTCGCTGAAAAACT
	*recA*	ACGCTGCTGATCTTCATCAACCAGATCCGTATGAAAATTGGTGT
GDCS^a^ MLST (Pasteur) [16]	*dinB*	CTGCGCGATATCCCTATTGCTATTGGCGGCAGCCGCGAACG
	*icdA*	GCATTCCTGCAACAGATCCTGCTGCGTCCGGCTGAATATGATGTTATCGCCTGTATGAACCT
	*pabB*	GAAAATCTGATGATTGTCGATTTAATGCGTAATGATATCGGTCG
	*polB*	AAACATTGCCTGCCGGAGATTGTGACTAACATCTGGCACGGGCGCGATGAAGCCAAACG
	*putP*	GGGATTGTAGTGTTTAGTTTGCTGGGTAAAGCGCCGTCAGCGGCGATGCAAAAA
	*trpA*	TTTGGTATTTCCGCCCCGGATCAGGTAAAAGCAGCGATTGATGCAGGAGCTGCGGGCGCGATTTC
	*trpB*	AAAGAGCAGCTACTGGTGGTTAACCTTTCCGGTCGCGGCGATAAAGACATCTTC
	*uidA*	GAACTGAACTGGCAGACTATCCCGCCGGGAATGGTGATTACCGA
*Campylobacter* MLST [18]	*aspA*	CAAATTTCAGGTGTTTTAAAACGTGTTGCAACAAAACTTTCTAAAGTATGTAATGACTTAAGACTT
Synthetic construct, MyIC [19] GenBank Accession: FJ357008	My-IC 1	GATCAGCTACGTGAGGTCCTACGACGATCGCCAAGCATGCCCTAGCTAAGATGCATCGATTGCTCATCACGT
	My-IC 2	ACGTTAGGTCGACTAGGAGGACTGGAGTGCATCGACTAGCTAAGATGGTTCGATTGCTCATCACGAAGGTTAG

^a^ Genomically Dispersed Conserved Sequences (GDCS)

### Simulation of sequencing reads

The ART software [20] was used to generate “synthetic” Illumina reads from the approximately 5.5 Mbp closed genome of *Escherichia coli* O157:H7 Sakai (EC20040078, GenBank accession NC_002695) [21]. Datasets of synthetic reads were generated in triplicate for twelve different lengths to determine the impact of read length on target identification. Since the ART software does not allow users to choose the number of reads to be generated, we determined depth of coverage values to be used in conjunction with each read length to keep the number of reads constant by adjusting the theoretical depth of coverage, *D*, using eq 1:

$$D_{adj}=C•\frac{L_{r}}{50}\left(\frac{N_{r}•L_{r}}{S_{g}}\right)$$ (1)

Where *D* corresponds to the expression defined in parentheses i.e., the number of reads, *N*_*r*_, generated during a sequencing run times the length, *L*_*r*_, of said reads divided by the size of the genome sequenced, *S*_*g*_ (viz. 5,498,430 bp). *D*_*adj*_, the adjusted depth of coverage, is obtained by adjusting *D* as a function of the ratio of the read length tested over the maximum read length studied (50 nt), and normalizing the number of reads to 0.5, 1, 1.5 and 2 million (M) by dividing 1M by the number of reads produced with a theoretical fold coverage of 10 (i.e., 1,099,686 bp, S1 Table). The adjusted depth of coverage and the read length were used as ART input. Similarly, synthetic reads were created in triplicate for 12 depth-of-coverage values to determine the impact of this parameter and k-mer length on target identification using 21-nt reads (S2 Table). Simulations were also performed with laboratory data. Raw FASTQ files from three sequencing runs of *E*. *coli* O157:H7 Sakai EC20040078 (OLC-1042) previously performed in our laboratory were trimmed to 21 nt using FASTX Trimmer (v. 0.0.13.1) from the FASTX-Toolkit [22].

### e-probe mapping

Synthetic reads from each dataset were mapped to the e-probes using the Sequence Mapping and Alignment Tool (SMALT) (v. 0.7.4) [23] with a sampling step size of 1. Multiple word lengths (k = 5, 7, 9, 11, 13, 15, 17, 19 and 20 nt) were compared and a k-mer size of 5 was ultimately selected for the GeneSippr application. Using programs from the SAMtools suite (v. 0.1.19-44428cd) [24], e-probe sequences were indexed (faidx) in preparation for the creation of variant call files, while mapping files were sorted (sort), indexed (index), and converted to variant call format (VCF and BCL2VCF). Base calls were extracted from VCF files using custom scripts. All custom scripts are available at: https://github.com/OLC-LOC-Bioinformatics/geneSippr. The percentage of sequence identity, corresponding to the proportion of e-probe bases mapped by the simulated reads, was determined using in-house Perl and R scripts and averaged for all probes (Mean Percent Identity; MPI). *E*. *coli* O157 was the only serotype used in the modeling, as the genome of *E*. *coli* O157:H7 Sakai (EC20040078, GenBank accession NC_002695) [21] was used to simulate the reads. E-probes were deemed to be accurately identified when a minimum of 55% of the sequence was mapped by at least 2 sequencing reads. All computational analyses were performed using Bio-Linux 8 on 2 x Intel Xeon CPU X5650 processors (12 cores at 2.66MHz) with 192 GB RAM.

### Bacterial strains

A variety of *E*. *coli* strains with defined serological characteristics and virulence gene profiles were used to evaluate the performance of the GeneSippr (Table 2). The STEC strains used in this study were previously described [25]. Two strains of *Enterobacter cloacae*, a species commonly co-isolated with STEC and two strains of generic *E*. *coli*, were used as negative controls. Bacteria were routinely grown on nutrient agar (Difco, Becton, Dickinson & Co., Sparks, MD) as previously described [4].

**Table 2:** Strains and results of GeneSippr analysis.

**Strain #**	**Strain (profile)** ^a^	**O26**	**O45**	**O103**	**O111**	**O121**	**O145**	**O157**	***stx***_*1*_	***stx***_*2*_	***eae***	**QC**	**gDNA (ng)** ^b^	**Number of reads (M)** ^c^	**Sequencing depth** ^d^
EDL 933	*E*. *coli* O157:H7 (*stx*_*1*_,*stx*_*2*_,*eae*)	-	-	-	-	-	-	+	+	+	+	15	3.0	1.6	6.9
EC20040078/ Sakai	*E*. *coli* O157:H7 (*stx*_*1*_,*stx*_*2*_,*eae*)	-	-	-	-	-	-	+	+	+	+	15	19.2	1.9	7.9
OLC-464	*E*. *coli* O26:H11 (*stx*_*1*_, *eae*)	+	-	-	-	-	-	-	+	-	+	15	42.8	2.8	11.9
OLC-683*	*E*. *coli* O26:H11 (*eae*)	+	-	-	-	-	-	-	-	-	+	15	212	0.1	0.6
OLC-731	*E*. *coli* O26:H11 (*stx*_*1*_,*stx*_*2*_,*eae*)	+	-	-	-	-	-	-	+	+	+	15	201	3.5	14.7
OLC-716	*E*. *coli* O45:H2 (*stx*_*1*_, *eae*)	-	+	-	-	-	-	-	+	-	+	15	41.6	2.5	10.5
OLC-975	*E*. *coli* O45:H23	-	+	-	-	-	-	-	-	-	-	15	17.8	2.2	9.4
OLC-679	*E*. *coli* O103:H2 (*stx*_*1*_, *eae*)	-	-	+	-	-	-	-	+	-	+	15	44.6	2.2	9.4
OLC-728	*E*. *coli* 0103:H11 (*stx*_*1*_, *eae*)	-	-	+	-	-	-	-	+	-	+	15	167	1.0	4.4
OLC-455	*E*. *coli* O111:H11 (*stx*_*1*_, *eae*)	-	-	-	+	-	-	-	+	-	+	15	41.3	1.8	7.4
OLC-715*	*E*. *coli* O111:NM (*stx*_*1*_,*stx*_*2*_,*eae*)	-	-	-	+	-	-	-	+	+	+	15	286	0.3	1.1
OLC-682	*E*. *coli* O111:NM (*eae*)	-	-	-	+	-	-	-	-	-	+	15	132	4.9	20.6
OLC-710	*E*. *coli* O121:H19 (*stx*_*2*_,*eae*)	-	-	-	-	+	-	-	-	+	+	15	39.6	3.0	12.8
OLC-791	*E*. *coli* O121:NM (*stx*_*2*_,*eae*)	-	-	-	-	+	-	-	-	+	+	15	208	3.3	13.9
OLC-675	*E*. *coli* O145:NM(*stx*_*1*_, *eae*)	-	-	-	-	-	+	-	+	-	+	15	39.0	3.1	12.9
OLC-684*	*E*. *coli* O145:NM (*eae*)	-	-	-	-	-	+	-	-	-	+	15	245	0.3	1.1
OLC-469	*E*. *coli* O157:H7 (*stx*_*1*_,*stx*_*2*_,*eae*)	-	-	-	-	-	-	+	+	+	+	15	70.2	5.2	21.6
OLC-797	*E*. *coli* O157:H7 (*stx*_*1*_,*stx*_*2*_,*eae*)	-	-	-	-	-	-	+	+	+	+	15	40.7	2.5	10.4
OLC-1470	*E*. *coli* O157:H7 (*stx*_*1*_,*stx*_*2*_,*eae*)	-	-	-	-	-	-	+	+	+	+	15	8.3	1.8	7.4
OLC-733	*E*. *coli* O85:H1 (*stx*_*2*_)	-	-	-	-	-	-	-	-	+	-	15	46.8	0.8	3.4
OLC-816	*E*. *coli* O104:H7 (*stx*_*2*_)	-	-	-	-	-	-	-	-	+	-	15	121	1.6	6.5
OLC-721	*E*. *coli* O113:H21 (*stx*_*2*_)	-	-	-	-	-	-	-	-	+	-	15	171	4.1	17.0
OLC-1051	*E*. *coli* O128:NM (*stx*_*1*_)	-	-	-	-	-	-	-	+	-	-	15	32.4	3.7	15.7
OLC-732	*E*. *coli* O177:NM (*stx*_*2*_,*eae*)	-	-	-	-	-	-	-	-	+	+	15	99.6	6.0	25.4
OLC-1547	generic *E*. *coli*	-	-	-	-	-	-	-	-	-	-	15	41.3	2.1	9.0
OLC-1555	generic *E*. *coli*	-	-	-	-	-	-	-	-	-	-	15	13.0	2.8	11.7
OLC-1682	*Enterobacter cloacae*	-	-	-	-	-	-	-	-	-	-	5	15.1	1.4	6.1
OLC-1683	*Enterobacter cloacae*	-	-	-	-	-	-	-	-	-	-	5	2.9	2.1	8.6

^a^Based on strain characterization/CHAS results in previous work [4]

^b^Total genomic DNA isolated from a single colony

^c^Number of reads generate (in millions)

^d^Estimated fold coverage of genome achieved with 21-nt reads

*low coverage was observed for three strains in one run. Analysis was repeated every hour until QC targets were identified. For strains OLC-683, OLC-715, and OLC-684, QC and virulence targets were identified at cycle 125, cycle 175 and cycle 41, respectively.

### Extraction of genomic DNA and sequencing

For the GeneSippr analysis, isolates (Table 2) were cultured on nutrient agar (Difco, Becton, Dickinson & Co) overnight (14–16 hrs) at 37°C, and genomic DNA was extracted from single colonies using the Maxwell 16 Cell LEV DNA Purification kit (Promega, Madison, WI). DNA was quantified using the Quant-iT High-Sensitivity DNA Assay Kit (Life Technologies Inc., Burlington, ON). Sequencing libraries were constructed from 1 ng of gDNA using the Nextera XT DNA sample preparation kit (Illumina, Inc., San Diego, CA) and the Nextera XT index kit (Illumina, Inc.). Genomic sequencing of eight multiplexed samples was performed on the Illumina MiSeq Platform (Illumina, Inc.) using a 300 cycle MiSeq Reagent kit v2 or a 600 cycle MiSeq Reagent kit v3 (Illumina, Inc.). Paired-end sequencing was conducted with 21 base reads generated from the first strand and 281 (300 cycle kit) or 581 (600 cycle kit) base reads from the second strand.

### Early sampling of sequencing reads

In the experiments to determine the performance of real-time analysis of WGS data, base call (BCL) files were copied from the sequencing instrument following 37 cycles of sequencing. Files were then de-multiplexed and converted to FASTQ files using Illumina’s BCL2FASTQ conversion software (v. 1.8.3).

### Whole-genome mapping and gap analysis

Simulated and trimmed 21-nt reads, and reads derived from the GeneSippr analysis were mapped to the *E*. *coli* O157:H7 Sakai closed genome (EC20040078, GenBank accession NC_002695) [21] using SMALT with a word length of 5 and sampling step size of 1. The percent sequence identity was determined as detailed above using the full genome instead of e-probe sequences. Sequencing gaps and frequencies, as well as depth of coverage were determined from the BCL files using custom Perl and R scripts.

### Genome assembly and detection of full length virulence markers

Following completion of the sequencing run, sequencing errors in reads were corrected using Quake (version 0.3 with a k-mer size of 15) [26], and *de novo* whole-genome sequence assemblies were generated using SPAdes v. 3.1.1 [27]. Detection of a comprehensive set of full length virulence genes in assembled genomes was performed using the VirulenceFinder tool provided by the Center for Genomic Epidemiology (www.genomicepidemiology.org) [11]. Web-tools from this site were also used for multilocus sequence typing (MLST) of isolates.

## Results

The main goal of this project was to determine whether a WGS approach to STEC colony identification and characterization could be completed within a typical food microbiology laboratory working day, which is the time frame for the EHEC-7 CHAS method currently used in food testing laboratories at the Canadian Food Inspection Agency [4]. Typical MiSeq sequencing runs, which aim to generate the longest possible paired-end reads, facilitating genome assembly, consist of 300 to 600 reaction cycles that take more than two days to complete. To deploy sequencing as an alternative same-day test procedure in a food testing scenario it will be necessary to significantly reduce the time required to achieve pathogen identification to be in line with the current approach. A reduction in sequencing time would necessitate the use of shorter reads, while maintaining the accuracy of the identification of the target organism. To compare the WGS approach to the EHEC-7 CHAS, we used the same probe sequences, presently referred to as e-probes (Table 1). The operational parameters for the GeneSippr were defined on the basis of modelling experiments designed to 1) determine the minimum read length required for accurate mapping of reads to e-probe sequences (eliminating false positives), and 2) determine the genome coverage needed to ensure the absence of large gaps encompassing the target e-probes (i.e., avoiding false negatives).

### Modeling Target Detection

**Fig 2:**
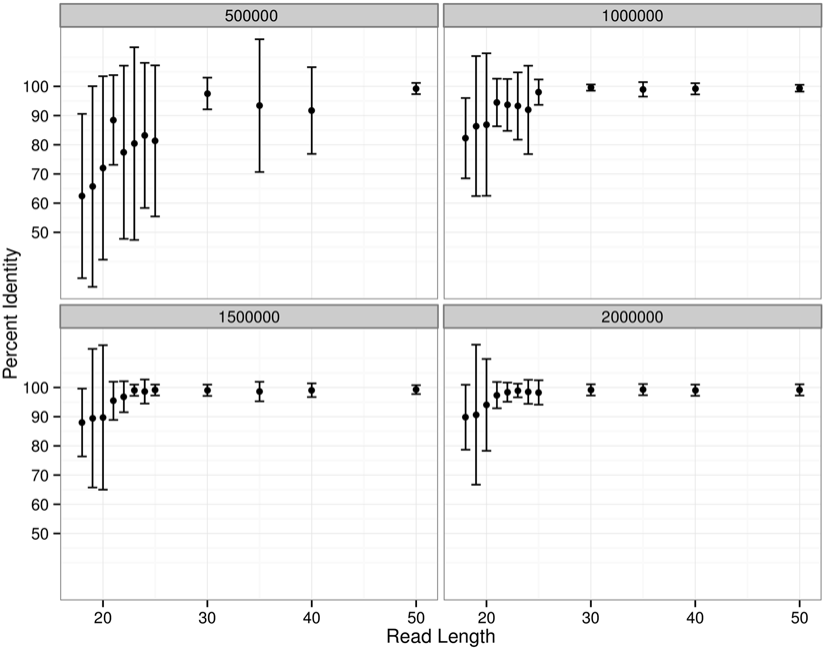
Minimum read length required for accurate identification of target sequences. The genome of *E*. *coli* Sakai (EC20040078) was used to randomly generate triplicate datasets of 0.5M (top left), 1M (top right), 1.5M (bottom left) and 2M simulated reads (bottom right) for twelve read lengths ranging from 18 to 50 nt (144 datasets). The reads from individual datasets were then mapped to the target sequences, and the mean percentage of sequence identity (MPI) was calculated for each dataset. The average and standard deviation of the three mean percentage of sequence identity obtained for each dataset are shown. An MPI above 90% was used as the threshold for accurate identification.

**Fig 3:**
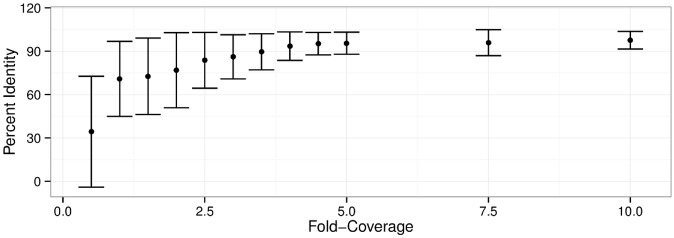
Impact of coverage depth on identification of target sequences using 21-nt simulated reads. The genome of *E*. *coli* Sakai (EC20040078) was used to randomly generate triplicate datasets of simulated 21-nt reads for twelve depths of coverage (36 datasets). The reads from individual datasets were then mapped to the target sequences, and the mean percentage of sequence identity (MPI) was calculated for each dataset. The average and standard deviation of the three MPI values obtained for each dataset at different depths of coverage are shown. An MPI above 90% was used as the threshold for accurate identification.

Synthetic Illumina sequencing reads, generated using the genome of *E*. *coli* O157:H7 Sakai (EC20040078), were mapped to the e-probe sequences to determine the impact of read length, number of reads, depth of coverage, and mapping k-mer size on the identification of target sequences. The mean percent identity (MPI), which corresponds to the average proportion of e-probe bases covered by the reads, was used as a measure of identification. As the reads were generated from this reference genome, perfect coverage of a given e-probe would be expected to generate 100% identity, and any lower identity figure would indicate lack of coverage of the bases within that target. An average MPI ≥ 90% was arbitrarily chosen to serve as the threshold for accurate target detection. The EHEC-7 CHAS and 15 genomically dispersed, conserved sequences (GDCS) sequences were consistently detected using reads as short as 21 nt from the datasets containing 1 M reads or more (Fig 2 and S1 Table), whereas the datasets of 500,000 synthetic reads failed provide enough data to adequately map target sequences using reads under 30 nt. The same mapping approach revealed that a depth of coverage as low as 3.6 was sufficient to identify targets using 21-nt reads (Fig 3 and S2 Table). Taken together, these results indicate that GeneSippr requires a minimum of approximately 880,000 reads to accurately identify target sequences mapping to the e-probes using 21-nt reads. Sequencing runs generating fewer reads could nevertheless be used for target detection, but would require proportionately longer reads, and therefore more time. The size of the k-mer seed used during the mapping of 21-nt reads was shown to have little effect on MPI: k-mers of length 5 to 13 generated MPI above 90% using dataset reads corresponding to depths of coverage above 2.5 (S1 Fig and S2 Table). The negative control targets were not detected (0% MPI) in any of the simulations performed (data not shown).

**Fig 4:**
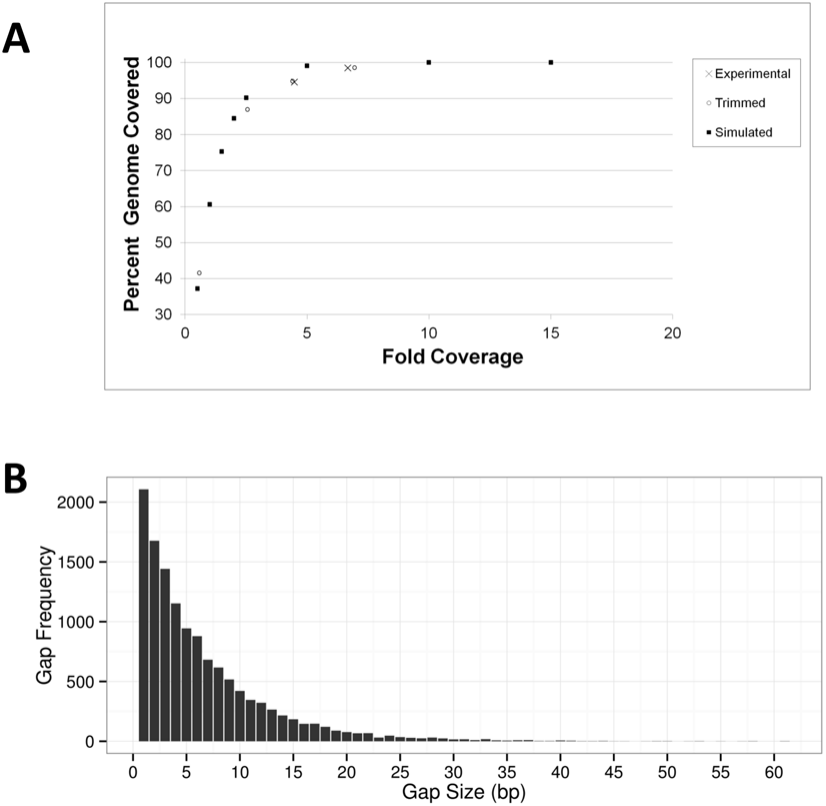
Comprehensive coverage of the *E*. *coli* Sakai reference genome with 21-nt reads. **A.** Mapping of 21-nt sequencing reads to the *E*. *coli* Sakai (EC20040078) reference genome indicates that a depth of coverage of 4 ensures that >95% of the genome is covered. The 21-nt reads sampled in real time (×) during two sequencing runs, reads trimmed to 21 nt (open circle) from two experimental runs (and the result of combining the two), and simulated 21-nt reads were mapped against the closed *E*. *coli* Sakai (EC20040078) reference genome. The percentage of the reference genome covered by the reads from each sequencing run and simulations are shown. **B.** Mapping of 21-nt experimental reads sampled in real time to the *E*. *coli* Sakai reference genome demonstrates the absence of significantly large gaps. The 21-nt reads sampled in real time from the sequencing run that provided the best genome coverage were mapped against the *E*. *coli* Sakai reference genome (EC20040078) and gaps of all sizes (bp) were counted using a custom script. Frequency of each gap size is indicated. No gaps larger than 61 bases were observed.

To determine whether a sequencing run consisting of 21-nt reads would adequately cover the entire genome without leaving significant gaps, synthetic reads were mapped to the single ~5.5 Mb contig of the closed *E*. *coli* O157:H7 Sakai EC20040078 reference genome. The percentage of genome bases covered by the reads was used as a measure of the breadth of coverage (Fig 4A). In order to assess whether the mapping of synthetic reads was comparable to experimental data, we trimmed 250-nt reads from three sequencing analyses of our *E*. *coli* O157:H7 Sakai strain (OLC1042) to 21 nt, and mapped the individual datasets to the same reference genome. The proportion of the genome covered by the synthetic reads was comparable (within 5 percent) to the coverage obtained from reads derived from laboratory data, indicating that the simulations accurately represented experimental conditions. We also combined the trimmed reads from the two best datasets to evaluate the benefit of sequencing redundancy and achieved a proportion of genome covered of 98.5% at 7-fold depth of coverage, indicating that sequencing duplicates during an urgent outbreak investigation can provide greater assurance of whole genome representation.

### GeneSippr design

The parameters for the execution of the GeneSippr protocol were chosen on the basis of the results obtained in the modeling experiments and in previous STEC sequencing runs. In constructing the sequencing library, reads from each isolate are identified by unique 8-nt index sequences tagged onto each end of the DNA fragments. These indices are processed by the MiSeq instrument following sequencing of the first strand of a paired-end sequencing run (i.e. after 150 to 300 cycles, depending on the sequencing kit used), significantly delaying sequencing data processing. To reduce the time required to complete the index determination process, paired-end reads were generated asymmetrically, with 21-nt reads of the first strand followed by 281- or 581-nt reads of the second strand. To ensure adequate coverage of each isolate with 21-nt reads, a maximum of 8 isolates were multiplexed in each test run. Following the creation of FASTQ files from the primary output base-call files, 21-nt reads were retrieved and subsequently mapped to the e-probe sequences, while the sequencing run continued to completion. The outputs of this analysis included 1) determination of presence or absence of the 10 EHEC-7 genomic markers (identification of priority STEC isolate), and 2) a quality metric indicating the validity of the foregoing determination through analysis of the GDCS markers. This quality metric operates on the premise that detection of all 15 GDCS markers at the time of sampling the sequence data during its generation indicates that information covering the entire genome was available to support positive and negative assessments of the EHEC-7 targets (Table 1). A set of three negative control e-probes (i.e., sequences not expected to occur in the *E*. *coli* genome) was also included to test for non-specific detection of genetic markers (Table 1). Isolates for which GDCS e-probes were not identified could be re-analyzed every 10 additional cycles until the sequencing run was completed.

### Analysis of STEC strains by GeneSippr

The performance of GeneSippr with respect to the identification of the target EHEC-7 gene markers in STEC strains having different genotypes [4] was verified using single colonies from a panel of target and non-target bacteria grown on agar plates (Table 2). The entire GeneSippr protocol provided for the identification of STEC colonies within a total time frame of 9 hrs. The time course for the entire procedure starting with a colony isolate on an agar plate was as follows: gDNA was extracted and quantified within one hour, followed by construction of libraries and their preparation for sequencing within three hours, retrieval and analysis of sequence data from the first read after 37 cycles of sequencing (5 hours) (Fig 1). The bioinformatic component of the GeneSippr process was completed within 5 minutes. Using this procedure, greater than 5-fold coverage was achieved for most of the colonies sequenced using either the 300 or 600 cycle sequencing kits.

Genomic DNA (gDNA) was purified from single colonies from a panel of 19 priority STEC strains (five O157:H7, three O26, two O121, three O111, two O45, two O103 and two O145), and an assortment of seven other *E*. *coli* strains plus two *Enterobacter cloacae* isolates with an average yield of 85.6 ng/colony (Table 2). In general, sequencing libraries were constructed using 1 ng of gDNA, with the exception of two samples (OLC-791 and OLC-1683) for which only 0.5 ng of gDNA could practically be used. Yield of genomic DNA did not impact subsequent analyses.

The gene markers corresponding to the EHEC-7 e-probes were correctly identified for each priority STEC strain as the GeneSippr results were in complete agreement with the EHEC-7 CHAS determinations performed separately on the strains (Table 2). In addition, GeneSippr correctly identified the presence of *stx* and *eae* markers in a small set of non-priority STEC strains (*E*. *coli* O104:H7, O177:NM, O85:H1, O113:H21 and O128:NM). None of the EHEC-7 markers were identified in two strains each of generic *E*. *coli* and *E*. *cloacae*. These results demonstrate the reliability of GeneSippr in identifying the presence of the EHEC-7 markers in genomic DNA extracts from single colony isolates. Each of the 15 GDCS markers was identified by mapping to their respective e-probes in all of the *E*. *coli* strains. The ability to detect all of the GDCS markers in the *E*. *coli* strains underscores their suitability as indicators of comprehensive genome sampling for this organism. Only five of these markers (*adk*, *icd*, *icdA*, *recA* and *trpB*) were detected in the *E*. *cloacae* strains despite adequate depth of coverage (5.7- and 8.2-fold) indicating that, as expected, not all of these sequences are present in non-target organisms. In any event, failure to detect all 15 GDCS markers in a given sample preparation would be indicative of either inadequate genome sampling or lack of sequence homology with the GDCS e-probes, flagging samples for further bioinformatic analysis. Lower coverage of 0.6–1.1 fold was observed for three strains in one run, due to the low numbers of clusters generated in the run for these strains. In these samples, GDCS could not be detected at the early time point, and, following the start of sequencing from the second strand, data from the instrument was manually retrieved after every 10 cycles and the GeneSippr procedure was repeated until GDCS were detected at cycle 41 (OLC-684), cycle 125 (OLC-683) and cycle 175 (OLC-715), after which the strains were correctly identified. The negative markers were not detected in any of the strains tested at any level of coverage.

A potential risk of the GeneSippr analysis is that significant gaps in sequencing coverage could encompass target genes, resulting in false negatives, even if all GDCS markers could be identified. To evaluate the presence of gaps in the sequence, the 21-nt reads generated by two sequencing runs generated from a culture of the *E*. *coli* EC20040078 strain from the Sakai outbreak [21] were mapped against the reference genome (GenBank accession NC_002695) to determine the proportion of the genome that was covered in the GeneSippr analysis, as described above for synthetic and trimmed reads (Fig 4A). The proportion of the reference genome covered by the reads from the two runs were 95% and 98.5%, obtained at depths of coverage of 4.4 and 6.7 folds respectively, which falls within 2% of the simulation data (approximated to 97% and 99.5% by linear interpolation between the bracketing data points). Moreover, no gaps greater than 61 bases were found in the sequencing run with the highest breadth of coverage (Fig 4B). No gaps larger than 102 bases were found in the other sequencing run (S2 Fig).

### Whole genome sequence analysis

To demonstrate that the sequence generated using the GeneSippr procedure was suitable for more comprehensive genomic analyses, following the completion of the sequencing run sequences were assembled *de novo*. Raw data and assemblies have been deposited at DDBJ/EMBL/GenBank under BioProject PRJNA273275. Accession numbers are listed in S3 Table. Coverage for the *E*. *coli* genomes ranged between 4.1 and 106.9 (S3 Table), with 67 to 402 contigs, with the exception of one low quality sample (OLC-683, S3 Table, asterisk). All of the full length virulence genes predicted to be in the STEC isolates by both the GeneSippr analysis and previous characterization by the EHEC-7 method [4] were detected (S4 Table). Full length MLST alleles were also identified for all but one sample (S3 Table). Note that even in the sample with the lowest depth of coverage (OLC-683, 2.2 fold), most of the full length genes were identified (S3 and S4 Tables).

## Discussion

The main premise of the GeneSippr approach is the analysis of raw read data during the early stages of the sequencing process in order to achieve same-day results in the identification of target marker sequences. The degree of coverage achieved at the time of data “sipping” is an important parameter to consider in judging the reliability of the procedure. While reads were short at the point of “sipping”, accurate detection of target genes was achieved. Both computer modeling and experimental results obtained in the analysis of a reference *E*. *coli* O157:H7 EC20040078 strain demonstrated that using 21-nt reads with a minimum of approximately 4-fold coverage resulted in sequence information being generated for more than 95% of all of the nucleotides and few sequence gaps. While this rapidly-generated, short-read data would generally be viewed as insufficient for a *de novo* draft genome assembly or for high-resolution analyses such as determining single nucleotide polymorphisms, our results obtained in the analysis of colonies of different STEC strains show that it is nonetheless adequate for reliable same-day determination of genome content such as the presence or absence of specific marker sequences, with comprehensive sequence data available in one or two days following completion of the sequencing run.

The GeneSippr approach was very robust, despite inconsistencies observed at initial stages in the laboratory procedure; specifically, the quantity of gDNA isolated from single colonies of bacteria was highly variable (Table 1), perhaps due to variations in the handling of colonies by different analysts. Nevertheless, this did not significantly impact the results of the GeneSippr analysis, even in cases where concentrations were so low that sequencing libraries were constructed using only 500 pg of gDNA instead of the 1 ng recommended by the Nextera XT manufacturer (Illumina, Inc.). This is consistent with the observation of Lamble *et al*. [28] that variability in gDNA concentration has minimal impact on the construction of transposon-based sequencing libraries. The fact that results were not impacted by a wide range of gDNA yields suggests that this technique is sufficiently robust for transfer to high-throughput testing laboratories. That said, lower coverage was observed for 3 strains in the final sequencing run conducted in this study and results for these strains were delayed (Table 2, S3 Table). To circumvent the delays associated with the occasional low quality sequencing library, sequencing libraries for critical samples could be prepared in duplicate.

All of the steps required to achieve the detection of STEC from a single colony were completed within a 9 h time frame, with sample preparation taking 4 h and the remaining time being required to complete 37 cycles of sequencing on the MiSeq instrument (Fig 1). It may be possible to improve the method workflow and the turnaround time for delivery of results with some minor modifications to the approach. For example, the method could be adapted to integrate an automated reporting feature so that analysts need not be on site to issue the report of analysis, which in a food safety investigation scenario could be quickly communicated to risk managers. Furthermore, the use of a single 8-base index sequence rather than the dual indices (16 bases) used presently would eliminate 8 cycles of sequencing, thereby reducing the analysis time by 40 minutes. Finally, more rapid methods for the isolation of gDNA from colonies, for example boil preparations, are currently being investigated.

While the goal of this project was to determine if accurate bacterial identification could be generated within a working day in a situation where timely results would be critical, it may be preferable to generate data that is suitable for draft genome assembly and more comprehensive genomic analyses at an early time point. This could be achieved by reducing the number of multiplexed strains and/or by increasing the length of the first read. Additionally, increasing the time devoted to bioinformatic analyses would enable detection of full length genes, and supplementary quality assurance targets. In the present study, data generated following the completion of the run were assembled and found to be suitable for MLST analyses and for detection of full length virulence genes (S3 and S4 Tables).

The timely generation and analysis of WGS information for isolates will not only be extremely valuable for their definitive identification, but also presents opportunities for risk profiling through the determination of other potentially relevant factors, such as virulence, antimicrobial resistance and typing markers. There may be instances where it is necessary to achieve more precise categorization of the risk attending a laboratory test result obtained in the context of a foodborne illness outbreak investigation. Furthermore, the definition of a pathogen or group of pathogens may change to reflect public health trends, for example, the STEC priority O serogroup designation may need to be adapted as new serogroups or strains emerge as significant public health risks [29–32]. Indeed, in 2011 the appearance of an STEC O104 strain as the causative agent of a major foodborne illness outbreak in Germany [29, 31, 33] took regulatory authorities by surprise as there were no detection methods available for this unanticipated event. Alternatively, the definition of “priority” STEC may shift from a serogroup basis to the use of virulence markers such as the Shiga-toxin type (and sub-type) and adhesins (e.g., *eae*, *aggR*, etc.).

In the GeneSippr approach, the number of markers that can be assessed is not limited by technical aspects associated with laboratory methods. GeneSippr can be configured as a highly multiplexed detection system, where new markers are added as the need arises. Conventional tools such as PCR do not allow such *ad hoc* determinations within the time course of a food safety investigation because of the need to optimize and validate each new primer added to a reaction system. Unlike wet lab techniques such as PCR, GeneSippr can be readily adapted at the bioinformatic level by designating a suite of appropriate e-probes for the diagnostic and quality control marker features. New markers can be “validated” *in silico* using extensive public and in-house WGS databases of target and non-target isolates. Such databases are continuously growing and being refined, providing an unprecedented wealth of contemporary genomic information with which to verify the suitability of diagnostic sequences, far exceeding the more traditional “wet lab” approaches in which PCR reagents are evaluated using limited bacterial culture collections. Likewise, the adaptation of GeneSippr to the detection of other pathogenic bacteria can be readily achieved provided that suitable e-probe sequences are available.

With the low level of multiplexing used in this study (8 isolates per sequencing run), the cost of sequencing a ~5 Mb organism such as *E*. *coli* was approximately $175 CDN per isolate. Increasing the number of multiplexed strains will reduce this cost. While this may appear to be more expensive than current PCR-based STEC detection methods [4], it is important to consider the total human and material resource requirements for all processes involved in completing a typical food investigation analysis (including delays incurred by shipping confirmed STEC isolates to reference laboratories for typing by serological, MLVA and PFGE techniques). Thus, all factors considered, the WGS approach has the potential to not only be faster but also less costly than standard methods. Furthermore, the adoption of a "one-test-fits-all" approach such as the GeneSippr will ultimately result in savings by reducing method development costs and training needs, simplifying quality assurance, and eliminating the need to maintain fresh reagents required to perform a multitude of pathogen-specific characterization methods.

Current molecular methods for the identification of pathogenic bacteria generally determine the presence or absence of key virulence genes (e.g., genes encoding toxins or colonization factors) using techniques such as PCR, which target short conserved sequences. Such an approach is limited in that it cannot determine the presence of a full-length functional gene, nor can it identify sequence variants with enhanced virulence. While the current version of GeneSippr does not target full length genes, reference mapping of the data derived in this procedure demonstrates that comprehensive coverage (>95%) of the genome is achieved at the early stages of the sequencing run. In future iterations of the method, data from positive isolates could be more fully analyzed to determine if virulence genes are likely to be functional, and to identify variants of virulence genes conferring higher risk [34–37]. It should also be noted that GeneSippr is intended to act as a two-stage process, in which the presence of genomic markers is discerned for the purposes of isolate identification during the early stages of the sequencing process, followed by completion of the sequencing to enable further detailed characterizations.

Comprehensive WGS data derived from the GeneSippr procedure conducted in front-line testing labs could also be used for high-resolution “DNA fingerprinting” of pathogens. Firstly, this would ensure that the strain analyzed can be unambiguously distinguished from in-house control strains, and secondly, data could subsequently be shared with public health stakeholders for molecular source tracking. Currently, PulseNet, the network of laboratories involved in molecular subtyping for outbreak detection, uses PFGE methods to type STEC isolates for source attribution purposes [8]. However, PulseNet investigators are currently evaluating the deployment of WGS-based approaches as an alternative DNA-based pathogen typing tool to PFGE [6, 8]. For highly clonal bacteria, such as *Salmonella enterica* serovar Enteritidis, WGS approaches have been shown to be advantageous since current typing methods (e.g. PFGE) sometimes lack the resolution required for detection of outbreaks, traceback and determination of transmission routes [5–7]. For such organisms, WGS approaches for molecular typing enhance outbreak detection by correctly associating epidemiologically linked isolates to outbreaks, while excluding genetically similar strains that are not associated with the cluster [5, 6]. In addition, use of the PFGE typing method at the Canadian Food Inspection Agency currently requires that strains be submitted to a secondary location for analysis. WGS information generated at food testing laboratories, through the GeneSippr, has the potential to be rapidly transferred to public health monitoring networks (e.g. PulseNet), thus eliminating significant delays, and biosecurity issues associated with shipping isolates. The GenomeTrakr Project led by the U.S. Food and Drug Administration provides a model of the deployment of sequencing capacity to front-line testing laboratories [6].

## Conclusions

The WGS approach to STEC typing described herein provided accurate genetic characterization of 26 *E*. *coli* strains within a time frame that is in line with current methods (Fig 1), providing a proof of concept for its utility in a food safety investigation context. To our knowledge this is the first demonstration of WGS implementation in the real-time detection of foodborne pathogens. The timeliness and ease of use of this method are comparable to the current EHEC-7 CHAS method used to detect 10 genetic markers of pathogenic *E*. *coli* from single colonies on primary isolation plates within a single working day [4], making it suitable for implementation in front-line testing laboratories supporting regulatory and industry food safety objectives.

The integration of real-time WGS capacity in food testing facilities would enhance capacity by improving the speed and accuracy of responses to existing and emerging threats in the food supply. The chief advantages of the GeneSippr approach include comprehensive characterization, elimination of the delays and biosecurity risks incurred by shipping pathogens to specialized facilities for typing, and ease of adaptation for new genetic targets. Automatic generation of reports could be integrated in the procedure to ensure that responsible individuals would be notified of test results in real time, as the data is generated. Given the impressive rate at which next-generation sequencing technology is evolving, it is reasonable to expect that the technologies underpinning the different elements of the GeneSippr process, such as DNA preparation, WGS platforms and bioinformatics tools will improve significantly in the next two-year period, lowering the cost and time frame for completing a WGS analysis even further. While we have investigated the use of this platform for food microbiology testing, this approach could be useful for any public health laboratory investigating pathogenic bacteria.

## Supporting Information

S1 Fig Target sequence identification is unaffected by k-mer length (5–13 nt) during mapping of 21-nt sequencing reads. The genome of *E*. *coli* Sakai (EC20040078) was used to randomly generate triplicate datasets of simulated 21-nt reads at 2-, 2.5-, 5- and 7,5-fold coverage (108 datasets). The reads from individual datasets were then mapped to the target sequences using nine k-mer sizes ranging from 5 to 20 nt, and the mean percentage of sequence identity (MPI) was calculated for each dataset. The average and standard deviation of the three MPI values obtained for each dataset at different k-mer sizes are shown. An MPI above 90% was used as the threshold for accurate identification.
(TIF)

S2 Fig Mapping of 21-nt experimental reads sampled in real time to the *E*. *coli* Sakai reference genome demonstrates the absence of significantly large gaps. The 21-nt reads sampled in real time from the sequencing run that provided the worst genome coverage were mapped against the *E*. *coli* Sakai reference genome (EC20040078) and gaps of all sizes (bp) were counted using a custom script. Frequency of each gap size is indicated. No gaps larger than 102 bases were observed.
(TIF)

S1 Table ART input values for read length and depth of coverage. (DOCX)

S2 Table ART input values for depth of coverage and resulting number of 21-nt reads in each dataset. (DOCX)

S3 Table WGS data generated by GeneSippr. To demonstrate quality of WGS data generated by the GeneSippr procedure, following completion of the sequencing run, reads were assembled *de novo*. Quality metrics and accession numbers for raw and assembled data are provided.
(XLSX)

S4 Table Identification of full length virulence genes in WGS assemblies derived from the GeneSippr procedure. WGS assemblies generated from GeneSippr data were queried using the VirulenceFinder tool available at www.genomicepidemiology.org. Full length gene identification confirms probe-based gene identification determined using GeneSippr and with the EHEC-7 laboratory method.
(XLSX)
