# Supplementary figures and images for: GeneSippr: A Rapid Whole-Genome Approach for the Identification and Characterization of Foodborne Pathogens such as Priority Shiga Toxigenic Escherichia coli
Source: PLoS One. 2015 Apr 10;10(4):e0122928. doi: 10.1371/journal.pone.0122928 (PMC4393293; doi:10.1371/journal.pone.0122928)

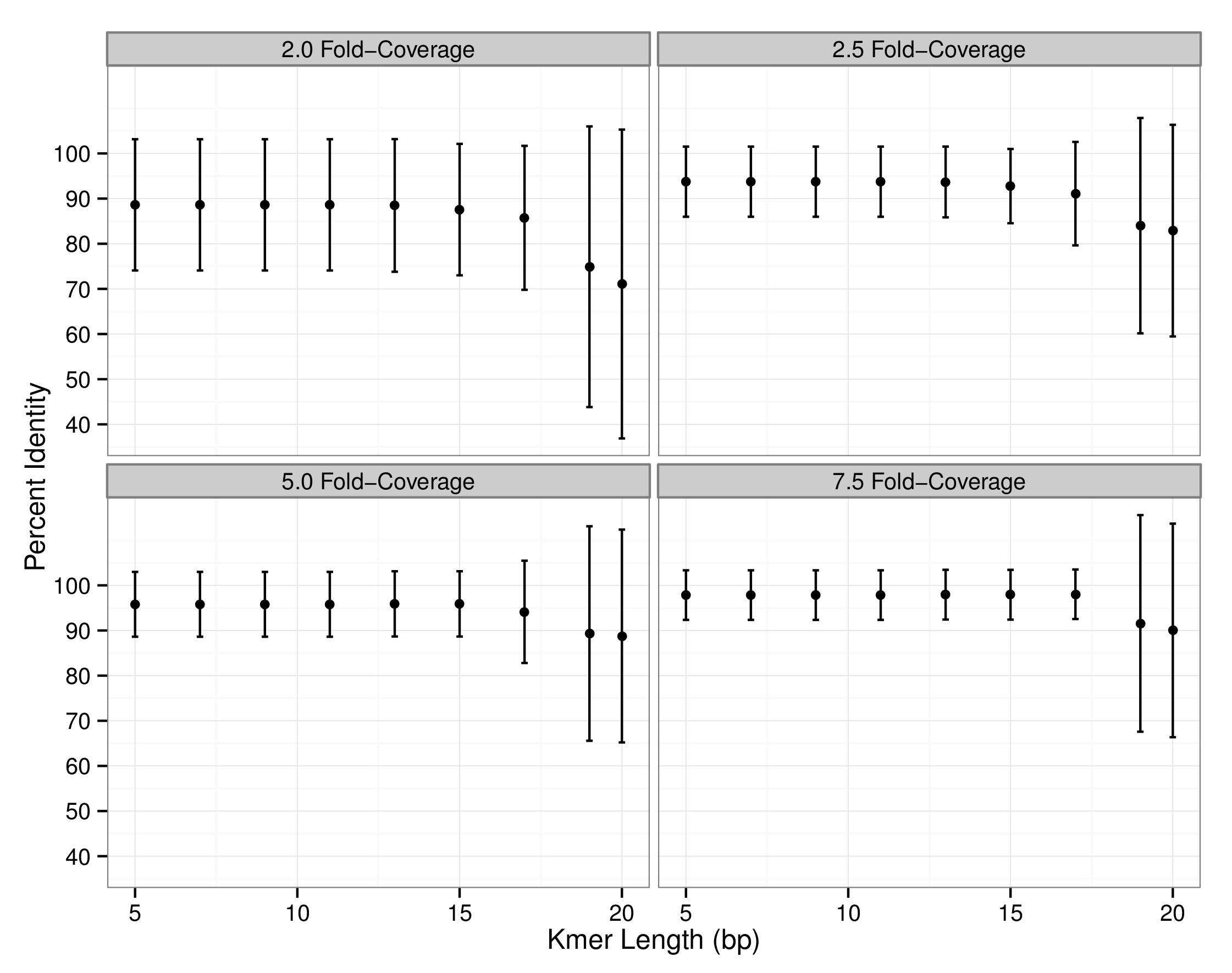

Supplement: S1 Fig — The genome of E. coli Sakai (EC20040078) was used to randomly generate triplicate datasets of simulated 21-nt reads at 2-, 2.5-, 5- and 7,5-fold coverage (108 datasets). The reads from individual datasets were then mapped to the target sequences using nine k-mer sizes ranging from 5 to 20 nt, and the mean percentage of sequence identity (MPI) was calculated for each dataset. The average and standard deviation of the three MPI values obtained for each dataset at different k-mer sizes are shown. An MPI above 90% was used as the threshold for accurate identification. (TIF) [file pone.0122928.s001.tif]

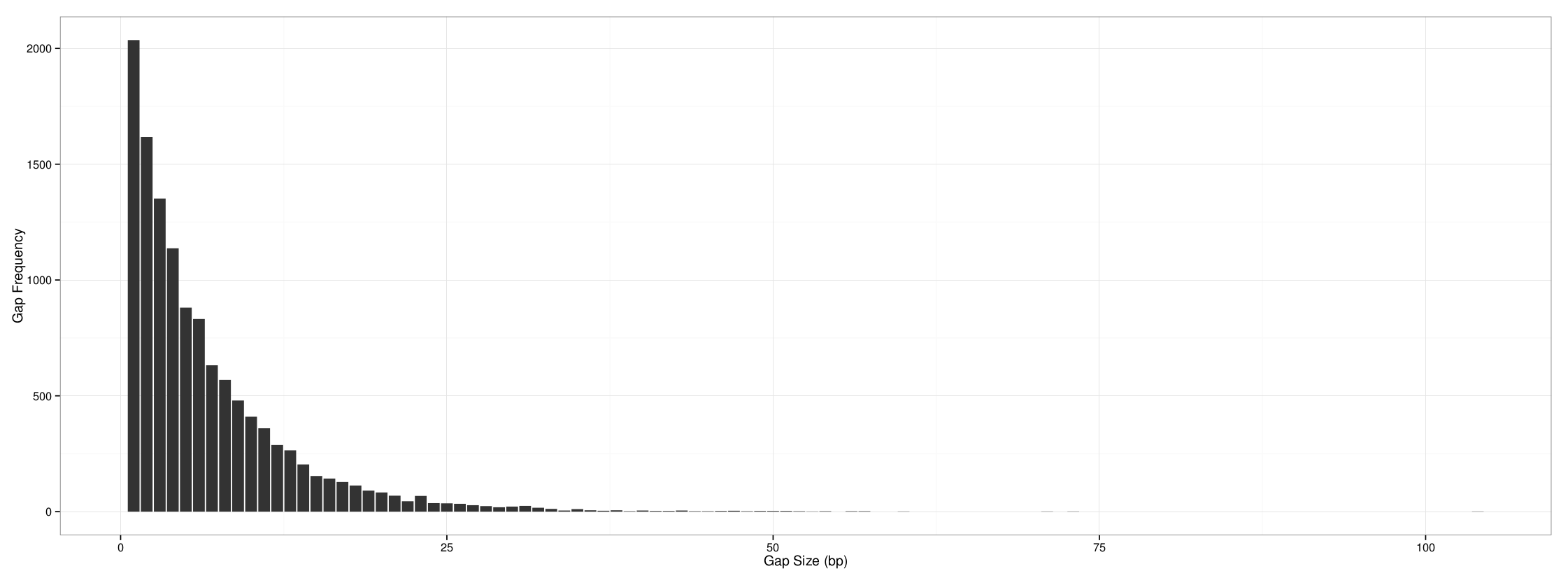

Supplement: S2 Fig — The 21-nt reads sampled in real time from the sequencing run that provided the worst genome coverage were mapped against the E. coli Sakai reference genome (EC20040078) and gaps of all sizes (bp) were counted using a custom script. Frequency of each gap size is indicated. No gaps larger than 102 bases were observed. (TIF) [file pone.0122928.s002.tif]
