# Supplementary material for: GeneSippr: A Rapid Whole-Genome Approach for the Identification and Characterization of Foodborne Pathogens such as Priority Shiga Toxigenic Escherichia coli
Source: PLoS One. 2015 Apr 10;10(4):e0122928. doi: 10.1371/journal.pone.0122928 (PMC4393293; doi:10.1371/journal.pone.0122928)
Supplement: S1 Table — (DOCX) [file pone.0122928.s003.docx]

**Table S1. ART input values for read length and depth of coverage.**

| *L_r_* nt^a^ | *D_adj_ N_0,5M_* ^b^ | *D_adj_ N_1.0M_* ^b^ | *D_adj_ N_1.5M_* ^b^ | *D_adj_ N_2.0M_* ^b^ |
| --- | --- | --- | --- | --- |
| 18 | 1.636831 | 3.273662 | 4.910493 | 6.547324 |
| 19 | 1.727766 | 3.455532 | 5.183298 | 6.911064 |
| 20 | 1.818701 | 3.637402 | 5.456103 | 7.274804 |
| 21 | 1.909636 | 3.819272 | 5.728908 | 7.638544 |
| 22 | 2.000571 | 4.001142 | 6.001713 | 8.002284 |
| 23 | 2.091506 | 4.183012 | 6.274518 | 8.366025 |
| 24 | 2.182441 | 4.364882 | 6.547324 | 8.729765 |
| 25 | 2.273376 | 4.546752 | 6.820129 | 9.093505 |
| 30 | 2.728051 | 5.456103 | 8.184154 | 10.91221 |
| 35 | 3.182727 | 6.365453 | 9.54818 | 12.73091 |
| 40 | 3.637402 | 7.274804 | 10.91221 | 14.54961 |
| 50 | 4.546752 | 9.093505 | 13.64026 | 18.18701 |

^a^Read length in nucleotides

^b^Adjusted depth of coverage values used as input in synthetic read simulation to obtain a constant *N_r_*, where *r* is the number of reads in millions, at various read lengths.
