# Supplementary material for: GeneSippr: A Rapid Whole-Genome Approach for the Identification and Characterization of Foodborne Pathogens such as Priority Shiga Toxigenic Escherichia coli
Source: PLoS One. 2015 Apr 10;10(4):e0122928. doi: 10.1371/journal.pone.0122928 (PMC4393293; doi:10.1371/journal.pone.0122928)
Supplement: S2 Table — (DOCX) [file pone.0122928.s004.docx]

**Table S2. ART input values for depth of coverage and resulting number of 21 nucleotide-long reads in each dataset.**

| *D* ^a*^ | *N_r_* 21nt^*^ |
| --- | --- |
| 0.5 | 130915.5 |
| 1 | 261831 |
| 1.5 | 392746.4 |
| **2** | **523661.9** |
| **2.5** | **654577.4** |
| 3 | 785492.9 |
| 3.5 | 916408.3 |
| 4 | 1047324 |
| 4.5 | 1178239 |
| **5** | **1309155** |
| **7.5** | **1963732.5** |
| 10 | 2618310 |

^a^ Theoretical depth of coverage values used as input in synthetic read simulation to obtain number of reads *N_r_* at a constant read length of 21 nucleotide.

^*^ Datasets in bold were also used to assess the effect of k-mer size on mapping accuracy.
